# A multi-band double-inversion radial fast spin-echo technique for T2 cardiovascular magnetic resonance mapping of the heart

**DOI:** 10.1186/s12968-018-0470-y

**Published:** 2018-07-19

**Authors:** Mahesh Bharath Keerthivasan, Sagar Mandava, Kevin Johnson, Ryan Avery, Rajesh Janardhanan, Diego R. Martin, Ali Bilgin, Maria I. Altbach

**Affiliations:** 1 Department of Electrical and Computer Engineering, University of Arizona, Tucson, AZ USA; 2 Department of Medical Imaging, University of Arizona, Tucson, AZ USA; 3 Siemens Medical Solutions USA, Inc., Tucson, AZ USA; 4 Department of Medicine, University of Arizona, Tucson, AZ USA; 5 Department of Biomedical Engineering, University of Arizona, Tucson, AZ USA

**Keywords:** Multi-band pulses, Radial, Fast spin echo, Black blood imaging, Double inversion recovery, T2 mapping

## Abstract

**Background:**

Double inversion recovery (DIR) fast spin-echo (FSE) cardiovascular magnetic resonance (CMR) sequences are used clinically for black-blood T2-weighted imaging. However, these sequences suffer from slice inefficiency due to the non-selective inversion pulses. We propose a multi-band (MB) encoded DIR radial FSE (MB-DIR-RADFSE) technique to simultaneously excite two slices. This sequence has improved signal-to-noise ratio per unit time compared to a single slice excitation. It is also motion robust and enables the reconstruction of high-resolution black-blood T2-weighted images and T2 maps for the excited slices.

**Methods:**

Hadamard encoded MB pulses were used in MB-DIR-RADFSE to simultaneously excite two slices. A principal component based iterative reconstruction was used to jointly reconstruct black-blood T2-weighted images and T2 maps. Phantom and in vivo experiments were performed to evaluate T2 mapping performance and results were compared to a T2-prepared balanced steady state free precession (bSSFP) method. The inter-segment variability of the T2 maps were assessed using data acquired on healthy subjects. A reproducibility study was performed to evaluate reproducibility of the proposed technique.

**Results:**

Phantom experiments show that the T2 values estimated from MB-DIR-RADFSE are comparable to the spin-echo based reference, while T2-prepared bSSFP over-estimated T2 values. The relative contrast of the black-blood images from the multi-band scheme was comparable to those from a single slice acquisition. The myocardial segment analysis on 8 healthy subjects indicated a significant difference (*p*-value < 0.01) in the T2 estimates from the apical slice when compared to the mid-ventricular slice. The mean T2 estimate from 12 subjects obtained using T2-prepared bSSFP was significantly higher (p-value = 0.012) compared to MB-DIR-RADFSE, consistent with the phantom results. The Bland-Altman analysis showed excellent reproducibility between the MB-DIR-RADFSE measurements, with a mean T2 difference of 0.12 ms and coefficient of reproducibility of 2.07 in 15 clinical subjects. The utility of this technique is demonstrated in two subjects where the T2 maps show elevated values in regions of pathology.

**Conclusions:**

The use of multi-band pulses for excitation improves the slice efficiency of the double inversion fast spin-echo pulse sequence. The use of a radial trajectory and a joint reconstruction framework allows reconstruction of TE images and T2 maps for the excited slices.

## Background

T2 weighted imaging is widely used in cardiovascular magnetic resonance (CMR) imaging protocols for the diagnosis of pathologies such as myocardial edema [1–8]. The double and triple inversion recovery fast spin-echo (FSE) pulse sequences are the choice for the generation of black-blood T2-weighted images [1, 2, 8]. The improved contrast between the myocardial tissue and the blood pool in these images enables better visualization of myocardial T2 signal variations associated with pathologies. Alternatively, T2 mapping approaches can be used to quantitatively characterize changes in T2 in tissues. Various methods based on FSE [9], gradient echo [10], and balanced steady-state free-precession (bSSFP) [5, 6, 11–13] pulse sequences have been proposed for myocardial T2 quantification.

Recently, we proposed [7] a method based on a double inversion recovery (DIR) radial fast spin-echo (RADFSE) sequence for T2 mapping of the heart. The use of a radial trajectory makes the acquisition motion robust [14–16] while also sampling the center of k-space at each echo time (TE). This allows the acquired data to be partitioned into undersampled k-space data corresponding to each TE [15] and the reconstruction of co-registered T2-weighted images with high temporal resolution. The DIR scheme used for black blood imaging, however, limits the scanning efficiency of the method due to the non-selective (NS) inversion pulse that is used to null the signal from blood. The NS pulse prevents the interleaving of multiple slices within the repetition time (TR), which leads to long dead times during the TR.

Various techniques have been proposed in the literature to overcome the slice limitation imposed by the DIR period and these typically involve the use of multiple slice selective (SS) inversions following the NS inversion pulse. In the method proposed by Song et al. [17] data are acquired from all the reinverted slices sequentially. Since each slice is acquired at a different TI, only one of the slices is at the null point of the blood signal. Parker et al. [18] proposed an approach for black-blood carotid imaging where following the reinversion of multiple slices on each TR, data acquisition from individual slices are interleaved across TRs. This method has the advantage that all the slices are acquired at the null point of blood. However, as the technique cascades data acquisition across TRs, the overall scan time increases linearly with the number of slices. This is a limiting factor in time-constrained applications like breath-held cardiac imaging. Using undersampled data reduces imaging time but at the expense of reduced signal-to-noise ratio (SNR).

In order to improve the slice coverage without loss in SNR, multi-band (MB) radio frequency (RF) encoding techniques such as Hadamard encoding [19] and phase offset multi-planar (POMP) imaging [20] have been proposed to achieve simultaneous multi-slice excitation. These methods modulate the RF pulses to spatially encode the magnetization along the slice dimension by imparting controlled phase offsets to the excited slices relative to each other and the individual slices are resolved by using this phase information to decouple the slices. These techniques have also been accelerated via parallel imaging by exploiting the spatial variation of coil sensitivities along the slice dimension [21].

In this work, we propose a multiband encoded DIR-RADFSE technique, MB-DIR-RADFSE, for simultaneous multislice dark blood imaging. By using Hadamard encoded MB RF pulses for excitation we can keep the same TI for all the slices ensuring they are acquired at the null point of blood. The encoded slices are jointly reconstructed using a subspace constrained algorithm to generate high resolution echo images and T2 maps for all the excited slices. By taking advantage of the properties of radial undersampling and the simultaneous slice excitation, the MB-DIR-RADFSE technique yields data for two slices with comparable image quality in the same amount of time required for a conventional single slice excitation. The technique is demonstrated using phantoms and in vivo cardiac imaging.

## Methods

### Pulse sequence and data acquisition

**Fig. 1:**
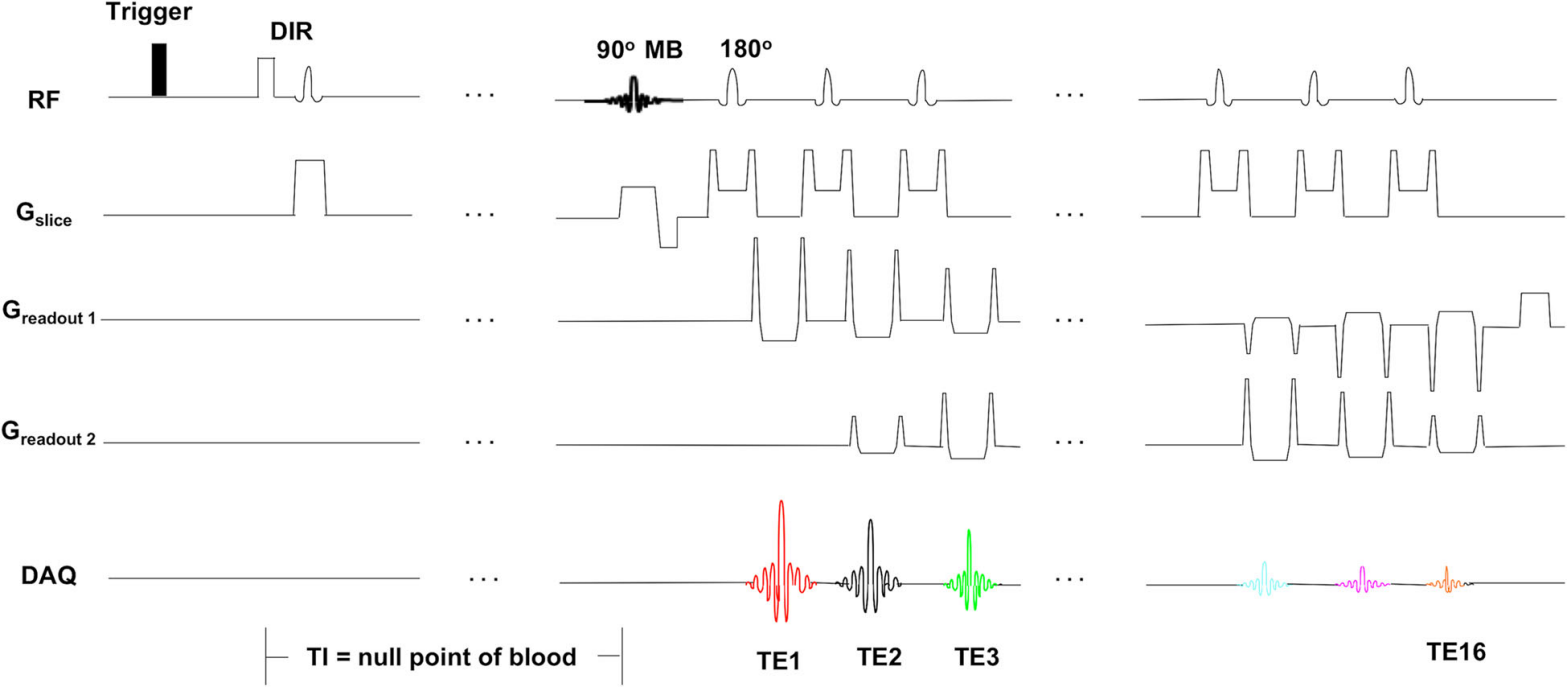
Pulse sequence diagram of the MB-DIR-RADFSE technique. The double inversion recovery (DIR) preparatory module, which comprises the non-selective (NS) and slab-selective inversion radiofrequency (RF) pulses, is played out following a cardiac trigger signal. After the TI chosen to null the signal from blood spins, data are acquired using an fast spin echo (FSE) readout with a radial scanning trajectory. The 90^°^ excitation is a multiband (MB) RF pulse designed to excite multiple slices; the refocusing pulses are all slab-selective to refocus the spins within the excited volume

The diagram of the MB-DIR-RADFSE pulse sequence is shown in Fig. 1. The sequence starts with a DIR preparation period consisting of a NS 180^o^ RF pulse followed by slab-selective 180^o^ RF pulse to re-invert the magnetization of spins in the slab-volume. The slab is selected to cover 2 times the volume of the imaging slices. At the null point of blood, determined by the selected TI, the imaging sequence is played out. The 90^o^ excitation is a MB RF pulse and the refocusing RF pulses are slab-selective to cover the excited volume. Data acquisition is performed using a radial scanning trajectory. For the CMR applications presented here the echo train length (ETL) was 16, allowing for the sampling of 16 different TEs.

**Fig. 2:**
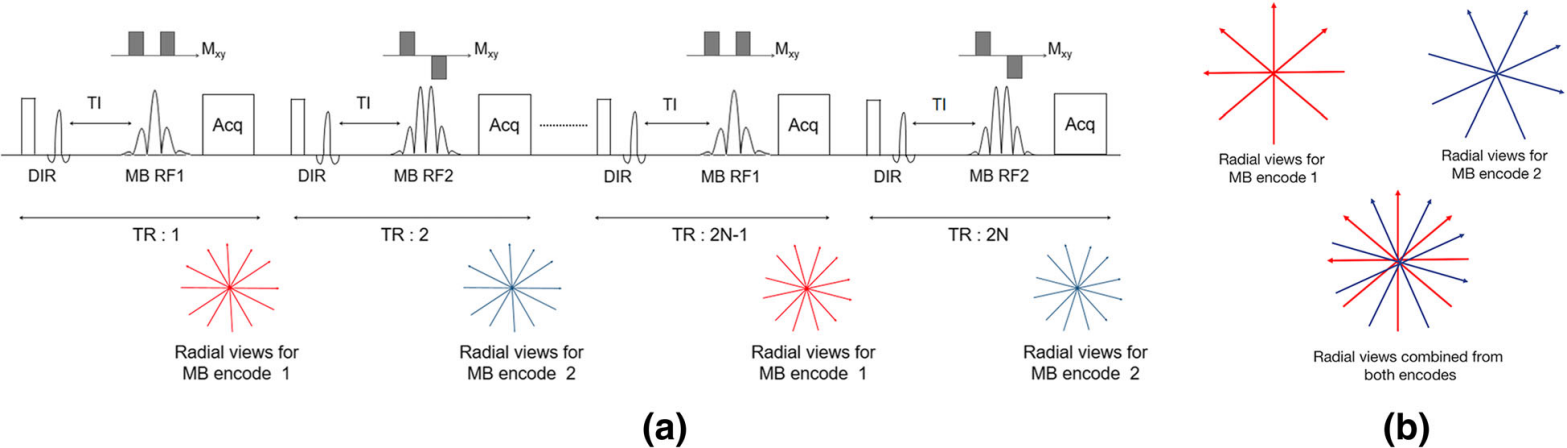
Schematic diagram showing the data acquisition for a 2-slice MB excitation. **a** The RF pulses for the two encodes are interleaved across TRs and radial views are acquired for each MB RF pulse. The radial views are rotated across the two encodes as shown in **b** to ensure better k-space coverage when the views from the different encodes are put together

The MB RF pulses are designed to excite multiple slices simultaneously. An RF phase encoding approach (Hadamard) is used to encode the signal in the slice dimension [19]. To achieve this, the MB pulses are cycled across TRs as illustrated in Fig. 2. The acquired radial views are rotated across the TEs and TRs in order to improve the k-space coverage (Fig. 2(b)). The slice crusher gradient moments were adjusted to suppress spurious signal from outside the excited slab due to imperfections in the modulation envelope.

### Image reconstruction from Undersampled radial data

Within a breath hold time, we can typically acquire 144–160 radial views per encode providing sufficient data to reconstruct an image for each slice with minimal artifacts due to spatial undersampling. This image (referred from now on as the “composite” image and used as the anatomical reference) contains radial views from all the TEs and has a T2-weighted contrast corresponding to the average of all acquired TEs. Since in radial FSE all views sample the center of k-space, images at the various TEs can be reconstructed from partial TE data sets generated from the same k-space data used to reconstruct the composite image.

Recently, our group proposed a model based reconstruction algorithm [22, 23] to reconstruct TE images from highly undersampled (4% relative to the Nyquist rate) RADFSE acquisitions. The algorithm approximates the underlying temporal signal variations using a lower dimensional principal component (PC) subspace of length *L* (*L* ≪ *ETL*) and recovers the individual PC coefficients using fidelity to the signal model and sparsity constraints.

In this work, we have extended the subspace constrained framework to reconstruct data acquired with simultaneous multislice excitation. The PC basis for each slice is generated by simulating the FSE signal using the slice resolved echo phase graph model (SEPG) [24] for a range of T2 and B1 values. This model accounts for imperfections in the slice profiles of the excitation and refocusing RF pulses, while also including effects of the crusher gradients and the preparation pulses. Each slice has a unique basis that is a function of the excitation and refocusing slice profiles. The individual excitation profiles are obtained by decoding the multiband RF pulses. The temporal signal for the *B* simultaneously excited slices are defined as ***X*** = [***x***_1_, ***x***_2_, …, ***x***_*B*_ ]^*T*^, where ***x***_*b*_ *for b* = 1, …, *B* is the temporal image with dimensions *ETL* × *N*,for an image with *N* pixels. The temporal variations in the image ***x***_*b*_ can be approximated by a *L* dimensional PC basis using ***x***_*b*_ ≈ ***ϕ***_*b*_***m***_*b*_ where ***ϕ***_*b*_ is the *ETL* × *L* dimensional PC basis for slice *b* and ***m***_*b*_ are the corresponding PC coefficients of dimension *L* × *N*. Using block matrix notation, the basis for all the excited slices are written as a diagonal matrix $$ \boldsymbol{\varPhi} =\left[\begin{array}{ccc}{\boldsymbol{\phi}}_1& \mathbf{\cdots}& \mathbf{0}\\ {}\mathbf{0}& {\boldsymbol{\phi}}_2& \mathbf{0}\\ {}\mathbf{\vdots}& \mathbf{\ddots}& \mathbf{\vdots}\\ {}\mathbf{0}& \mathbf{\cdots}& {\boldsymbol{\phi}}_B\end{array}\right] $$ and the PC coefficients are expressed as **M =** [***m***_1_, ***m***_2_…, ***m***_*B*_]^T^**.** This allows us to represent the temporal images for the different slices by ***X*** ≈ **ΦM =** [***ϕ***_1_***m***_1_, ***ϕ***_2_***m***_2_…, ***ϕ***_*B*_***m***_*B*_]^T^**.**

We define the k-space encoding operator for Hadamard excitation as **Ε** = ***FHS***. Here, ***S*** is the coil sensitivity matrix that modulates the temporal images from the different slices, ***H*** is the Hadamard encoding operator that maps the individual slices to the different encodes and ***F*** is the non-uniform fast Fourier transform operator corresponding to the acquired trajectory for the different echo times and multiband encodes. The forward signal model used to generate k-space can be written as, ***K*** = **Ε** ***X ≈*** **Ε ΦM**, where ***K*** is the multi-coil k-space corresponding to the temporal points for the different encodes. The reconstruction problem attempts to jointly recover the PC coefficients of all the excited slices and is formulated as:

1 $$ \widehat{\boldsymbol{M}}={\arg \min}_{\boldsymbol{M}}\kern0.5em {{\left|\left|\mathbf{E}\boldsymbol{\varPhi } \boldsymbol{M}-\boldsymbol{K}\right|\right|}_F}^2+\lambda\ R\left(\boldsymbol{M}\right)\kern6.75em $$

Here, the operator R() is a joint spatio-temporal regularizer achieved using the mixed l_2_/l_1_ norm [25] of the PC coefficients; *R*(*M*) = ||*ΨM*||_2, 1_. This norm is defined as ${\left|\left|\mathrm{M}\right|\right|}_{2,1}=\sum \limits_{\mathrm{n}=1}^{\mathrm{N}}{\left||{\mathrm{M}}^{\left(\mathrm{n}\right)}|\right|}_2$ where M^(n)^ is the n^th^ row of M and *Ψ* is the finite differences operator to promote sparsity. The regularization parameter *λ* was chosen based on the approach outlined in [22]. The individual echo images are reconstructed by projecting the PC coefficients onto the PC basis.

The optimization problem to reconstruct the PC coefficient images corresponding to the excited slices is solved using the alternating direction method of multipliers (ADMM) [26, 27]. The stopping criteria for the iterative algorithm was based on [25] and set to either a maximum of 50 iterations or when the residual error falls below a threshold of 5e^− 4^. All reconstructions were performed offline using MATLAB (MathWorks, Natick, Massachusetts, USA) and the Gadgetron library [28] on a workstation with a 3.6 GHz Intel Xeon processor E5–1620, 64GB RAM and an NVIDIA GeForce GTX 780 GPU. The reconstruction was implemented using a hybrid CPU-GPU approach; a CPU implementation of the ADMM algorithm was used where the non-uniform fast Fourier transform was computed on a GPU at each iteration. This resulted in a reconstruction time of about 22 min for a two-slice multi-band acquisition with 26 receiver channels.

T2 maps were generated from the TE images using a pattern recognition technique [29] that uses a pre-computed dictionary of curves based on the SEPG signal model. The T2 and B1 ranges for the subspace basis as well as the dictionary were 20–300 ms and 0.5–1.6, respectively. Since it has been shown that the SEPG model is insensitive to T1 [23, 24], T1 was fixed to 900 ms.

In order to determine the number of principal components, *L*, a Monte-Carlo simulation was performed to quantify the T2 estimation error from the subspace reconstruction. A digital MRXCAT heart phantom [30] was used to synthesize T2 maps corresponding to a mid-ventricular and apical slice using T2 values from the literature for the different tissue types. The T2 map was used to synthesize images at different echo times using the SEPG forward model, using ETL = 16 and echo spacing = 7.1 ms. The Hadamard operator was applied to the TE images corresponding to the two slices to generate the encoded data. Two-slice radial MB k-space data was generated by sampling the encodes using a radial re-ordering scheme with 160 views per encode and assuming coil sensitivities simulated using the Biot-Savart law [31]. A Monte-Carlo experiment was setup for 100 noise realizations and Gaussian noise was added to the real and imaginary components of k-space. The resultant data were reconstructed using the proposed reconstruction framework (Eq. 1) for different number of PC coefficients (*L = 2,3,4,5*). For each choice of *L*, the T2 map was estimated and the T2 estimation error compared to the ground truth was computed. The performance of the reconstruction was evaluated by computing the mean percent error in T2 estimation, $T{2}_{err}=\frac{\left|T2-\widehat{T2}\right|}{T2}\ast 100$, across the noise realizations.

### Phantom study

The MB-DIR-RADFSE pulse sequence was implemented on a 1.5 T CMR scanner (Aera, Siemens Healthineers, Erlangen, Germany).

Experiments were performed on a doped water phantom to adjust the power of the excite RF pulse in order to guarantee a flip angle of 90^o^ and to evaluate the SNR performance of the MB excitation. For this purpose, data at each TE point were acquired with sufficient radial views to ensure that there was no undersampling (512 radial views for 256 readout points) using MB-DIR-RADFSE. Images were reconstructed using a regridding algorithm. For comparison, reference data were acquired using single slice DIR-RADFSE with one and two averages. The experiment was repeated three times and the mean SNR was computed by measuring the mean signal and background standard deviation using multiple regions of interest in the object and the background.

In order to validate the T2 estimation accuracy, nickel-doped agarose gel phantoms were prepared using 1 mM NiCl_2_ and 4 different concentrations of agarose (3.6, 2.5, 1.5, 1%) to cover a T2 range of 35 ms to 130 ms with T1 of 930 ms. Reference T2 estimates were obtained from data acquired using a Cartesian single-echo spin-echo pulse sequence with acquisition matrix size = 128 × 64, TR = 5 s and 16 different TE values in increments of 10 ms. Data were acquired with MB-DIR-RADFSE and single slice DIR-RADFSE using the following parameters: field of view (FOV) = 21 cm, acquisition matrix = 256, readout bandwidth = 501 Hz/pixel, ETL = 16, echo spacing = 9 ms. A 60 bpm heart rate was simulated using a physiologic monitoring unit and the TR was set to 1RR. The single slice data were acquired with 160 radial views (scan time: 10 s/slice) and the MB data had 160 views per encode (scan time for two slices: 20 s). In order to compare the T2 mapping performance, data were also acquired using a vendor supplied T2-prepared balanced steady-state free precession (T2-prep bSSFP) pulse sequence [5] with the following parameters: FOV = 21 × 21 cm^2^, TR/TE = 2.5 / 1.23 ms, acquisition matrix = 192 × 123, GRAPPA factor = 2, flip angle = 70^o^, T2 preparation times = 0, 25, 55 ms.

### In vivo imaging

Written consent was obtained from subjects prior to imaging, in compliance with the Institutional Review Board requirements. The protocol was initially optimized on healthy volunteers and then added to the routine clinical CMR protocol.

Electrocardiogram-gated short-axis data were acquired using a 30-channel phased-array receiver coil using the single slice and two-slice MB-DIR-RADFSE pulse sequence with the following acquisition parameters: FOV = 36–40 cm, acquisition matrix = 256 points, readout bandwidth = 501 Hz/pixel, ETL = 16, echo spacing = 7.1 ms, and TR = 1RR. Spectral Attenuated Inversion Recovery (SPAIR) pulses were used for fat suppression and saturation bands were used to suppress unwanted signal from outside the FOV. Both SPAIR and saturation RF pulses were placed within the TI period and did not affect the scan time. The MB pulse sequence excited two 7 mm slices (21 mm slab with 7 mm inter-slice gap) with 160 radial views acquired per encode in a 18 s breath-hold.

Eight healthy subjects and 22 patients were scanned as part of this prospective study. The reproducibility of the two-slice MB-DIR-RADFSE method was evaluated in a subset of 15 subjects (5 healthy subjects and 10 patients) by imaging the subjects twice during the same scanning session. Late gadolinium enhancement (LGE) images were acquired for patients (as part of the clinical protocol) during the systolic phase of the cardiac cycle after the intravenous injection of MultiHance (Bracco S.p.A, Milan, Italy).

In a subset of 20 subjects (8 healthy subjects and 12 patients), data were also acquired using the T2-prep bSSFP sequence with a 7 mm thick slice. Other acquisition parameters for the T2-prep bSSFP pulse sequence were: FOV = 36 × 38 cm^2^, in-plane resolution = 2.61 × 1.98 mm^2^, GRAPPA factor = 2, TR/TE = 2.5 / 1.05, flip angle = 70^o^, T2-preparation times: 0, 25, 55 ms. Two slices were acquired for the healthy subjects to match the slice locations of the MB acquisition. For the patients, only one slice was acquired, matching one of the locations of the MB sequence.

### Data analysis

In order to quantify the efficiency of flow suppression in black blood imaging we computed the relative contrast (*C*_*rel*_) between the myocardium and the blood pool for the single slice and the MB pulse sequences. Relative contrast is defined using the signal in the myocardium (*Signal*_*MC*_) and the signal in the blood pool (*Signal*_*BP*_): *C*_*rel*_ = (*Signal*_*MC*_ − *Signal*_*BP*_)/*Signal*_*MC*_. The mean signal from a manually drawn region of interest (ROI) on the left ventricle (LV) and the blood pool was used for the calculations.

The spatial variability of the T2 estimates was studied by performing a segment analysis of the T2 maps. The T2 maps from the MB and T2-prep bSSFP acquisitions were segmented into the different myocardial sectors in accordance with [32].

A reproducibility study was performed to compare the T2 estimates obtained from two MB-DIR-RADFSE measurements. Mean T2 estimates were obtained from the LV ROI for the repeated acquisitions and Bland-Altman analysis was performed to assess the agreement of the T2 estimates. The reproducibility coefficient and the coefficient of variation were computed to determine the variation between the repeated measurements. The reproducibility coefficient is defined as 1.96 times the standard deviation of the difference in mean estimates and the coefficient of variation is the percentage standard deviation of the differences.

## Results

**Fig. 3:**
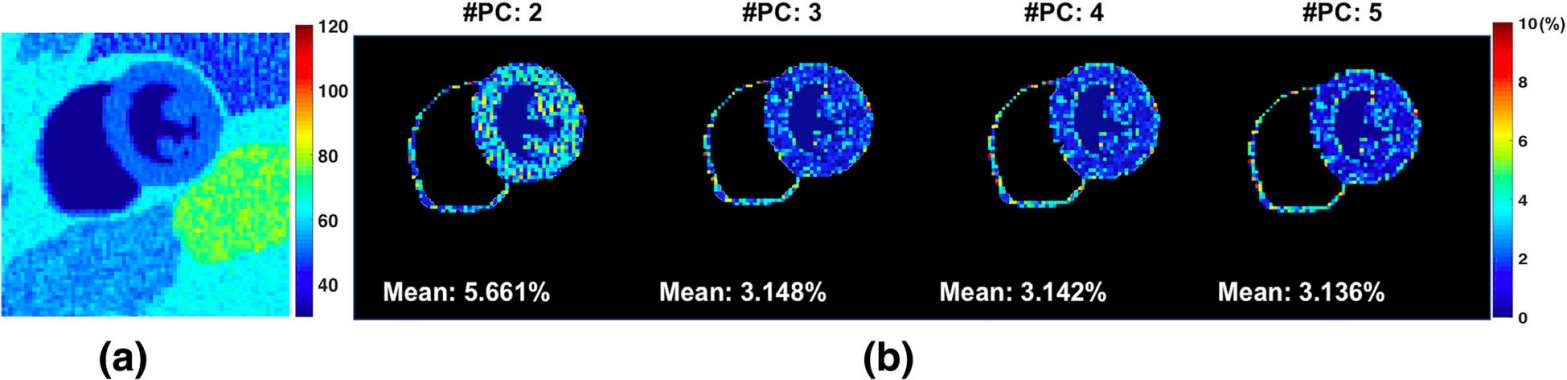
Effect of number of principal component coefficients on T2 estimation. **a** Ground truth T2 map obtained by simulating the MB-DIR-RADFSE pulse sequence using the MRXCAT phantom [31]. Monte Carlo simulations were performed to evaluate the T2 estimation error in terms of the number of principal components (PC) retained during reconstruction. **b** Percent error maps of the myocardium when data is reconstructed using 2,3,4, and 5 PCs. The mean percent error values are also indicated

Results of the Monte-Carlo simulation to study the effect of the number of PC coefficients on the T2 estimation error are shown in Fig. 3. The ground truth T2 map along with the percent error in the myocardium for the different number of PC coefficients is shown. Note that the average estimation error in the myocardial wall is comparable for PC coefficients > = 3. While simulations using noiseless data show that the model error decreases with larger *L* [22], the presence of noise leads to poor recovery of the TE images due to noise amplification [33]. As a tradeoff between model error and noise performance, we decided to use 3 PC coefficients for the reconstructions.

### Phantom study

The SNR efficiency of the MB excitation was evaluated using data acquired on the doped water phantom. The 2-slice MB excitation had a SNR of 32.8 a.u. and was comparable to a two-average single slice excitation (SNR = 33.5 a.u.) which was close to the theoretical √2 SNR increase compared to a single slice single-average excitation (SNR = 25.6 a.u.). These results indicate that the flip angle of the MB RF excitation was consistent with a 90^o^ excitation.

Data collected using the gel phantoms were used to verify the accuracy of the T2 mapping technique for MB excitation. The mean T2 estimates from the 2 slice excitation with MB-DIR-RADFSE are shown in Table 1 along with those from the single slice DIR-RADFSE and T2-prep bSSFP acquisitions. A two-tailed t-test was performed to compare the mean T2 values from the MB and the single slice DIR-RADFSE pulse sequences for the different phantoms. No significant difference was found between the means at *α* = 0.05. The T2-prep bSSFP pulse sequence over-estimated T2 values compared to the single-echo spin-echo reference and had significantly different (*p*-value < 0.03) means than MB-DIR-RADFSE. Since the vendor pulse sequence estimates T2 using a single exponential model, the T1/T2 effects present in the balanced SSFP pulse sequence are ignored resulting in estimation errors as reported in [34].

**Table 1:** T2 estimation accuracy

Reference T2 (ms)	Single slice DIR-RADFSE T2 (ms)	MB-DIR-RADFSE Slice 1 T2 (ms)	MB-DIR-RADFSE Slice 2 T2 (ms)	T2-Prep bSSFP T2 (ms)
37.6 ± 0.5	36.± 1.4	36.7 ± 1.3	36.4 ± 1.7	44.7 ± 1.5
51.7 ± 0.8	49.7 ± 1.9	50.5 ± 2.0	50.0 ± 1.4	57.8 ± 2.3
73.5 ± 0.9	72.6 ± 2.0	72.85 ± 2.19	72.3 ± 1.9	85.1 ± 3.0
127.1 ± 2.4	130.4 ± 4.1	130.6 ± 3.5	129.7 ± 3.8	146.1 ± 7.7

Mean and standard deviation of the T2 estimates on agarose gel phantoms are shown. The reference values were obtained from a single-echo spin-echo experiment as described in the Methods section. Note that the estimates from the proposed MB-DIR-RADFSE pulse sequence are closer to those from a single slice excitation scheme while the T2-prep bSSFP over-estimates the T2 values

### Black-blood imaging

**Fig. 4:**
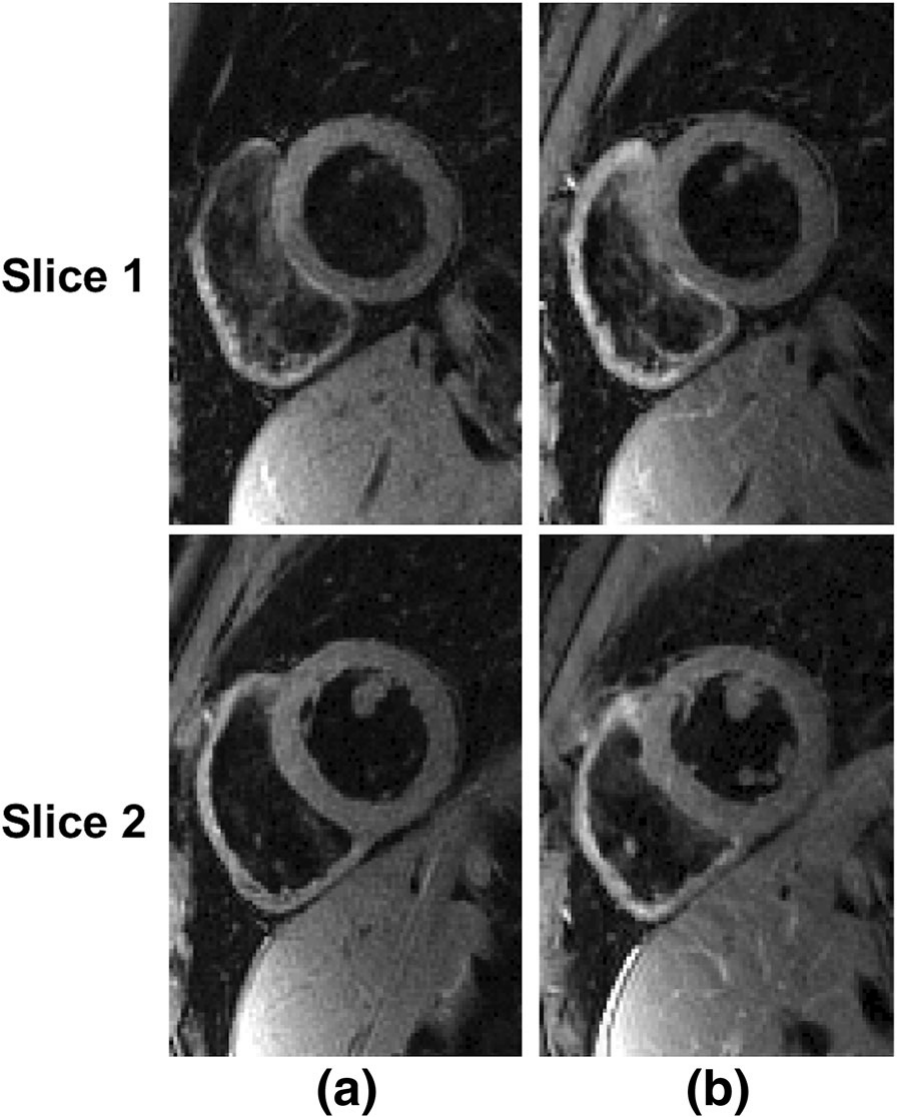
Single slice and MB-DIR-RADFSE comparison. Two short-axis slices acquired with **a** single slice DIR-RADFSE and **b** 2-slice MB-DIR-RADFSE. The multi-band slices have similar blood suppression to the single slice acquisition

Short-axis black-blood composite images of the heart reconstructed using all the acquired k-space views are shown in Fig. 4. Figure 4(a) shows images for two slices acquired using the single slice DIR-RADFSE pulse sequence; data acquisition per slice = 9 s. Figure 4(b) shows images, acquired at similar locations as those in 4(a), with 2-slice MB-DIR-RADFSE (data for the two slices were acquired in 18 s). Thus, the acquisition time for two slices with the single and multi-band DIR-RADFSE is the same (18 s) but the latter has the advantage of the of the √2 improvement in SNR as verified by the phantom experiments.

**Fig. 5:**
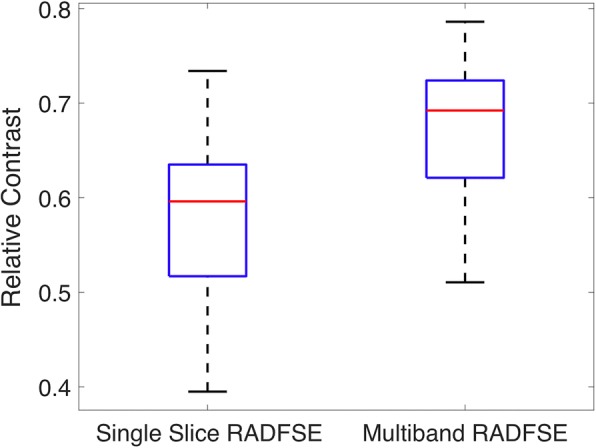
Box plot of myocardium-blood pool relative contrast. Relative contrast of the single slice and the multi band pulse sequences from data acquired in a subset of 15 subjects. Note that the relative contrast of the 2-slice MB-DIR-RADFSE is equal to or better than the single slice pulse sequence indicating comparable flow suppression when using multi band excitation

Since the two slices are excited simultaneously at the null point of blood, the signal from blood flow is well suppressed in both slices. Note that the flow suppression in both slices is comparable to that observed in the single slice DIR-RADFSE pulse sequence. The flow suppression was quantitatively measured for the single slice and the MB pulse sequences by computing the relative contrast of the myocardial tissue to the blood pool. The box plot in Fig. 5 shows the myocardium-blood pool relative contrast in a subset of 15 subjects demonstrating that the 2-slice MB-DIR-RADFSE technique has equivalent or better relative contrast than the single slice pulse sequence. It was observed that the difference in the myocardial signal between the MB and single slice pulse sequence was higher compared to the difference in the blood pool signal. This is because the MB excitation provides higher signal in regions where there is signal but does not affect regions of signal void (myocardium versus the blood pool). This resulted in a significantly (*p* < 0.01) higher relative contrast between the MB and single slice acquisitions.

### T2 mapping

**Fig. 6:**
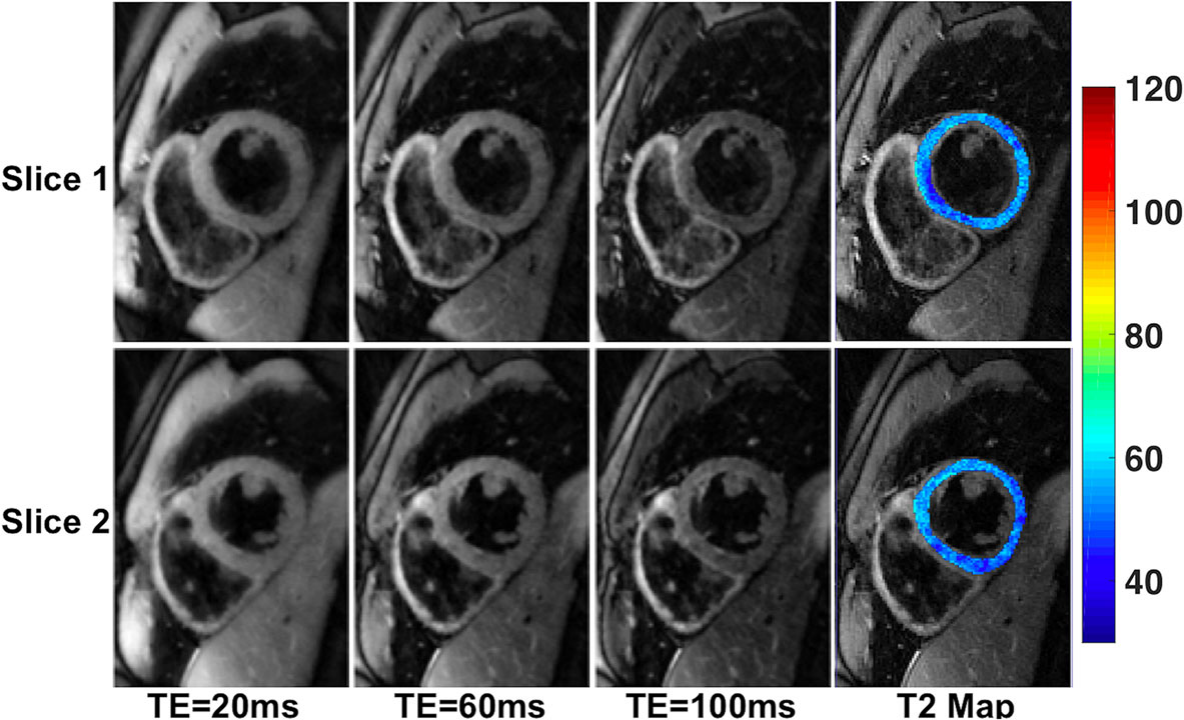
MB-DIR-RADFSE images of a healthy subject. Three out of the 16 TE images acquired using 2-slice MB-DIR-RADFSE on a healthy subject. The colorized T2 map of the LV is overlaid on the composite image. The mean T2 of the LV myocardial wall for the two slices are 52.5 ± 3.7 ms and 53.5 ± 3.5 ms

Since data were acquired using a radial k-space trajectory we can generate multiple TE images (number of TE images = ETL) for each slice. Three of the 16 TE images for each of the 2 slices acquired with MB-DIR-RADFSE on a healthy subject and reconstructed with the algorithm described in Eq. 1 using undersampled TE datasets (10 radial views per TE) are shown in the first three panels in Fig. 6. The figure also includes colorized T2 maps of the LV overlaid on the composite image. The mean T2 of the LV myocardium was 52.49 ± 3.71 ms and 53.45 ±3.51 ms for the two slices and these values fall within the normal range reported in the literature [5, 35, 36].

**Fig. 7:**
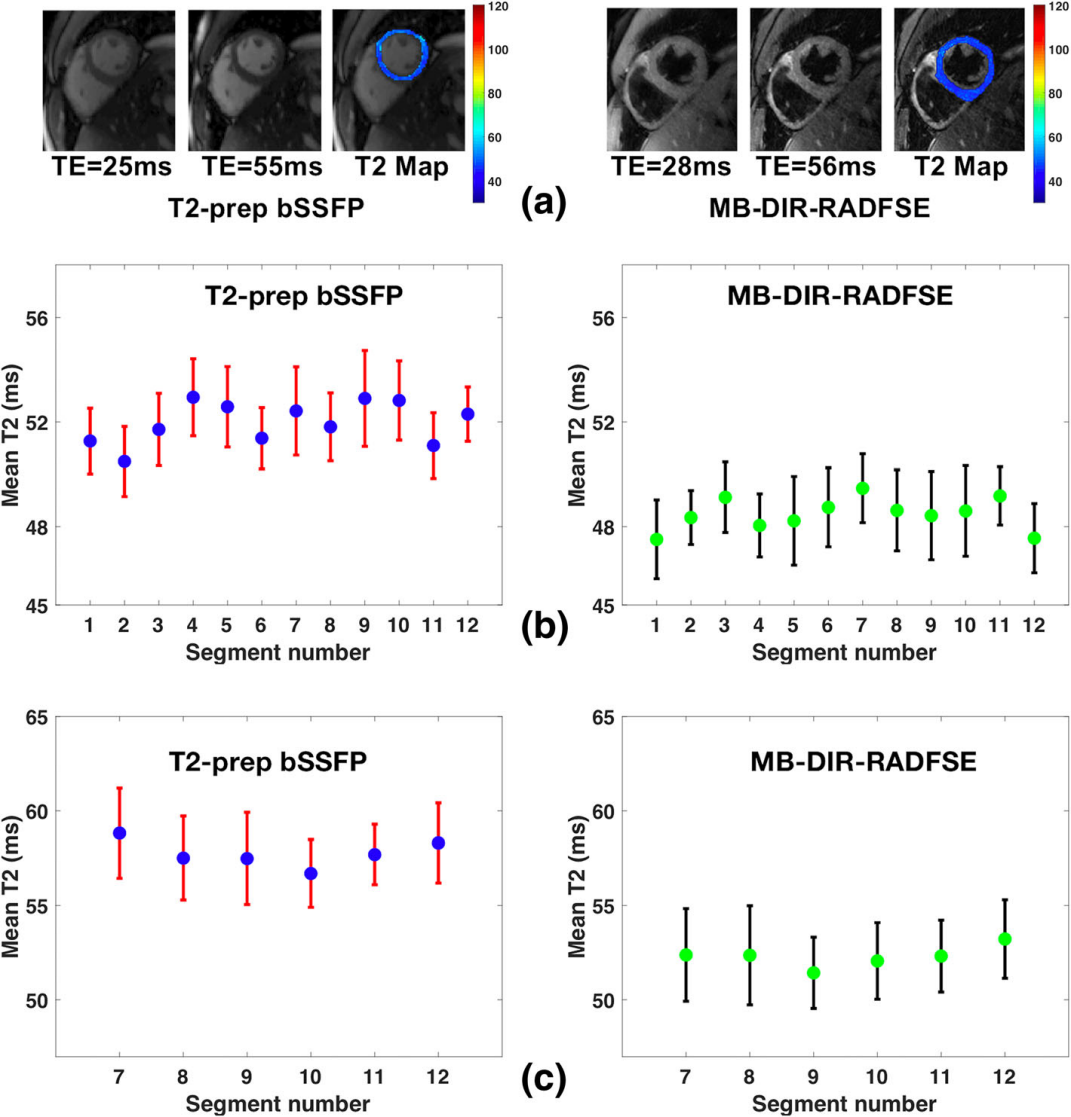
T2 estimates from MB-DIR-RADFSE and T2-prep bSSFP. **a** Representative TE images and T2 maps on a healthy subject. **b** Mean T2 values for 12 basal and mid ventricular segments averaged over 8 healthy subjectgs. **c** Mean T2 values for ventricular segments averaged over 12 clinical patients

T2 maps were acquired on 8 healthy subjects and 12 patients using both the T2-prep bSSFP and the MB-DIR-RADFSE pulse sequences. Figure 7a shows representative TE images and T2 maps acquired using the two pulse sequences on a healthy subject. Figure 7b shows plots of the mean T2 estimates across all healthy subjects for the 12 basal and mid-ventricular segments. The mean T2 values across all the 6 basal segments had a range of 48.6 ms - 53.3 ms and 45.6 ms – 49.6 ms for T2-prep bSSFP and MB-DIR-RADFSE, respectively. The mean T2 values across the mid-cavity segments varied between 48.4 ms to 53.3 ms for T2-prep bSSFP and from 45.9 ms to 50.1 ms for MB-DIR-RADFSE. Plots of the mean T2 estimates for the clinical patients are shown in Fig. 7c for both pulse sequences. T2-prep bSSFP has mean T2 values in the range of 56.7 ms – 58.8 ms and MB-DIR-RADFSE has values between 51.4 ms and 53.2 ms across the segments.

**Fig. 8:**
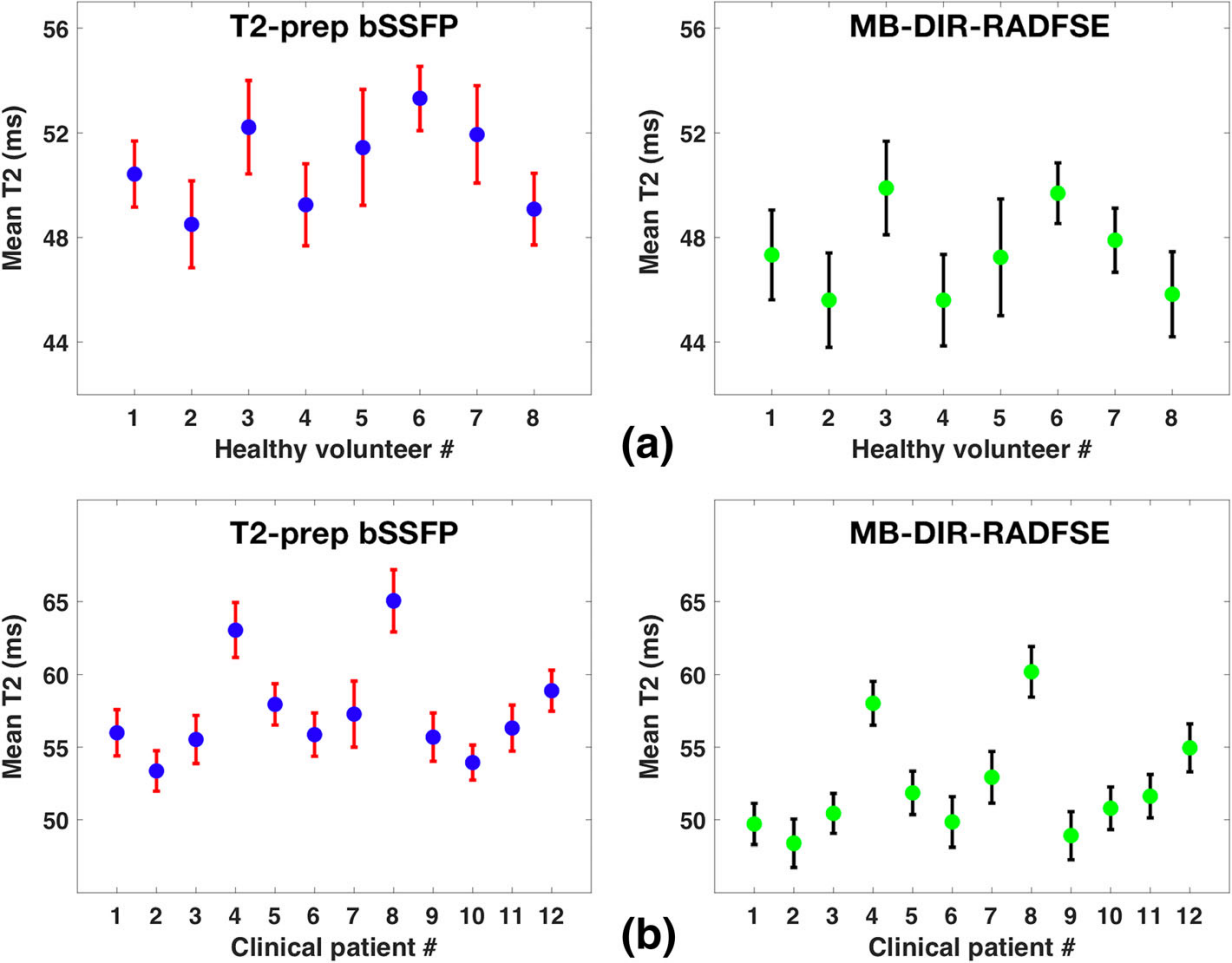
Mean T2 estimates across segments for healthy subjects and patients. Mean and standard deviation of T2 estimates over all myocardial segments obtained from MB-DIR-RADFSE and T2-prep bSSFP pulse sequences is shown for **a** 8 healthy subjects and **b** 12 patients

The mean T2 and standard deviation across all the segments for the healthy subjects and patients are shown in Fig. 8 for the two pulse sequences. The MB-DIR-RADFSE pulse sequence has a mean standard deviation of 3.39 ms across all segments averaged over the 8 healthy subjects. This was comparable to the mean standard deviation of the T2-prep pulse sequence (3.23 ms). The estimates from MB-DIR-RADFSE showed good agreement with T2-prep bSSFP, with a Pearson’s linear correlation coefficient of 0.922 and 0.958 for the healthy subjects and patients, respectively. Consistent with the phantom results, T2-prep bSSFP has a significantly (*p*-value = 0.012) higher mean T2 than the MB pulse sequence.

**Fig. 9:**
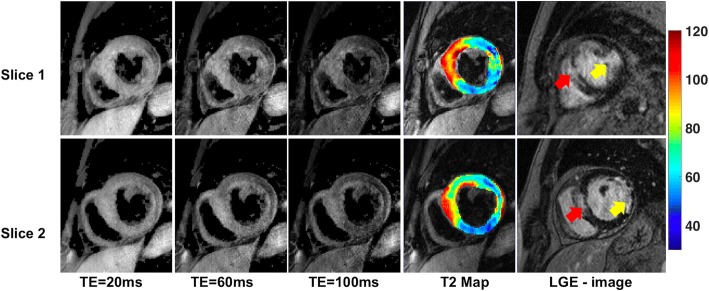
TE images, T2 maps and LGE images of a subject with acute myocardial infarction. The subject was diagnosed with infarcts in the anteroseptal wall (red arrow) and the lateral wall (yellow arrow). The infarct had a mean T2 of 94.8 ± 2.8 ms in the anteroseptal wall and 73.1 ± 6.4 ms in the lateral wall infarcts, respectively

TE images and T2 maps obtained from a 2-slice MB-DIR-RADFSE acquisition for a subject with a clinical presentation of acute myocardial infarction are shown in Fig. 9. According to the LGE image, the subject has two infarcts: one in the anteroseptal wall (red arrow) and another in the lateral wall (yellow arrow). The infarcts are characterized by the presence of near transmural hyper-enhancement on the LGE images which directly correlates with myocardial replacement fibrosis. The T2 maps for both slices show an increase in T2 relaxation times in the infarcted regions compared to the normal myocardium (58.7 ± 4.1 ms). However, the infarct in the anteroseptal wall has higher T2 values (94.8 ± 2.8 ms) than the one in the lateral wall (73.1 ± 6.4 ms) suggesting that the former is an acute infarct associated with myocardial edema related to inflammation produced by the recent infarct whereas the latter favors a chronic infarct due to the lack of significant edema. The estimated infarct T2 times are comparable to values previously reported [7, 31, 35].

**Fig. 10:**
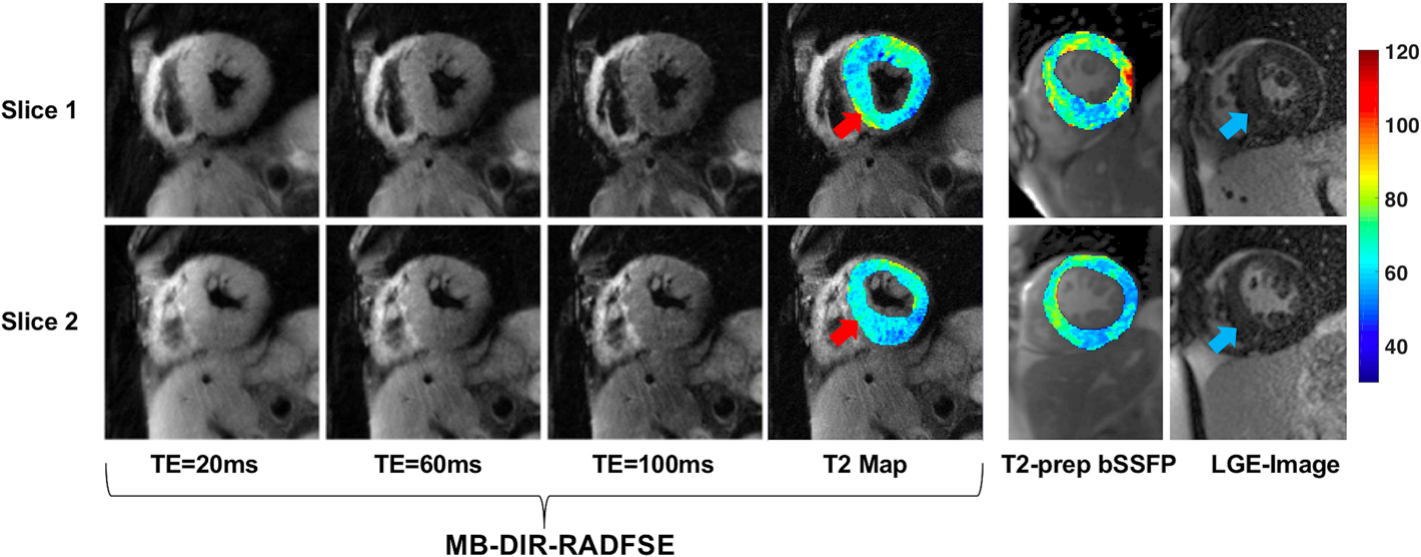
MB-DIR-RADFSE images from a subject with hypertrophic non-obstructive cardiomyopathy. Figure also shows T2 maps from T2-prep bSSFP and LGE images from similar but non-identical locations. T2 maps demonstrate diffuse abnormal T2 signal which was most pronounced in the septal wall (red arrows), correlating to patchy delayed enhancement on LGE images seen in the more hypertrophic septal segments (blue arrows)

Figure 10 shows an example of the MB-DIR-RADFSE technique (TE images and T2 map) performed on a subject with hypertrophic non-obstructive cardiomyopathy along with T2-prep bSSFP T2 maps and LGE images. The T2-prep bSSFP and LGE images were acquired at similar but non-identical slice locations. LGE imaging demonstrated patchy, diffuse delayed enhancement predominately involving the hypertrophied segments of the septum (blue arrows). When correlated to the T2 maps, the LV demonstrates diffuse hyper-intense T2 signal (> 60 ms in the MB-DIR-RADFSE and > 70 ms in the T2-prep bSSFP T2 maps) that is most pronounced in the area of septal hypertrophy with patchy delayed enhancement (red arrows). These findings suggest an area of myocardial edema related to fibrosis due to disorganized myocardial hypertrophy. Also, the T2 maps reveal additional areas of increased T2 (> 80 ms) in the lateral segments with no obvious area of fibrosis on the LGE image, suggesting areas of edema related to myocardial injury. These findings are consistent with recent reports [7, 37] which show areas of elevated T2 signal that are not co-localized to regions of fibrosis on the LGE images suggesting that T2 mapping may be a useful indicator of myocardial injury in hypertrophic cardiomyopathy.

### Reproducibility study

**Fig. 11:**
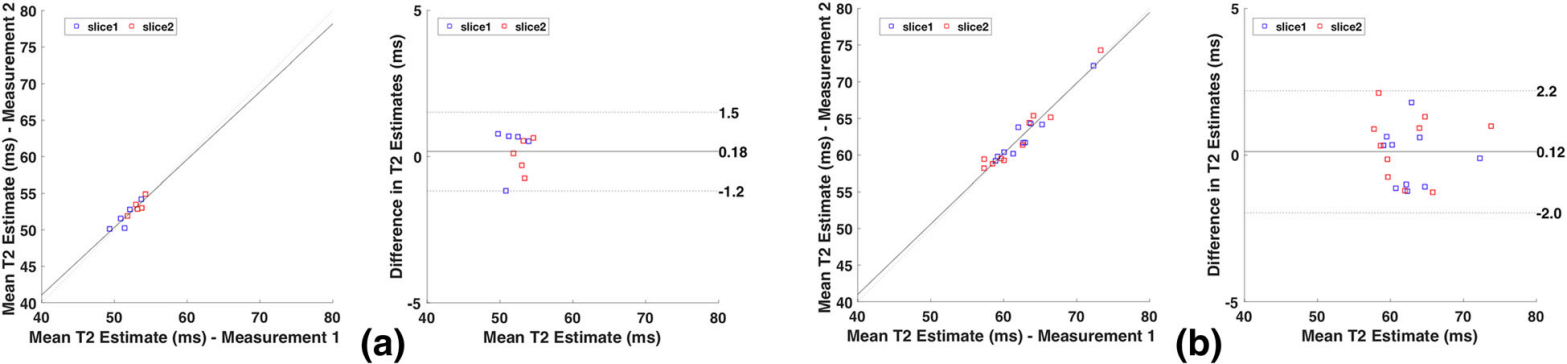
Bland Altman Analysis. Correlation of repeated MB-DIR-RADFSE acquisitions and corresponding Bland-Altman plot for **a** 5 healthy subjects and **b** 10 patients. The correlation plot indicates a significant correlation between the two sets of measurements for both the normal and patient groups with correlation coefficients of 0.90 and 0.968 respectively (*p*-value < 0.001). The dotted lines in the Bland-Altman plot represent 1.96 times the standard deviation of the differences in the estimates. The mean difference in T2 between the repetitions is 0.18 ms for the healthy subjects and 0.12 ms for the patients

Figure 11 shows the correlation and Bland Altman plots for both slices acquired with a 2-slice MB-DIR-RADFSE corresponding to the healthy subjects and patients. For the healthy subjects, the mean T2 difference was 0.18 ms with a coefficient of reproducibility of 1.34 and a 1.3% coefficient of variation indicating a high agreement in the mean T2 values from the two experiments. The mean T2 difference for the patient data was 0.12 ms with a coefficient of reproducibility of 2.07 and coefficient of variation 1.69%.

## Discussion

We have proposed a technique to simultaneously excite multiple slices using a DIR radial fast spin-echo pulse sequence, MB-DIR-RADFSE, for cardiac black-blood imaging. The use of a radial trajectory and a joint reconstruction framework allows the reconstruction of multiple TE images with high spatial and temporal resolution for each of the slices and the generation of T2 maps from data acquired as fast as a breath hold. This is an improvement over the single slice DIR-RADFSE method, recently published [7]; with MB-DIR-RADFSE, high-resolution whole heart T2 mapping can be achieved with half the number of breath holds (3–4 breath holds) compared to its single slice counterpart. T2 mapping enables the quantitative assessment of pathologies such as myocardial edema.

The radial acquisition has an advantage over the conventional Cartesian acquisition in terms of motion robustness. Radial acquisitions are sensitive to gradient delays leading to deviations of the acquired trajectory. These are corrected by measuring the gradient delays and using them to design the readout gradients [38]. In our scanner, the gradient delay calibration was performed once when the pulse sequence was implemented and applied to all experiments.

Compared to the T2-prep bSSFP the DIR-RADFSE methods (both single and MB) have higher spatial (1.4 × 1.4 mm^2^ vs 2.61 × 1.98 mm^2^) and temporal resolution (16 versus 3 TE points). The spatial resolution in SSFP pulse sequences is limited by the TR length; the temporal resolution is limited by the scan time, thus only a few TE times can be achieved within a breath hold. The limited number of TE points does not allowed full modeling of the signal evolution [33, 39]. This results in an over-estimation of the T2 values when using the T2-prep bSSFP vendor pulse sequence, as observed in Table 1. Also, in the T2-prep bSSFP, data for each TE set are acquired sequentially requiring spatial registration before T2 mapping.

The RF phase encoding approach used in this work requires the MB pulses to be switched across TRs to help separate the slices during reconstruction. This will lead to an overall increase in scan time proportional to the MB factor (N). In order to maintain the scan time to a reasonable breath hold duration, we take advantage of the √N boost in SNR in the 2-slice MB-DIR-RADFSE to increase the level of radial undersampling. The effect of radial undersampling in the TE images and T2 maps is minimized using a model-based reconstruction with spatial regularization. It should be pointed out that the minimum breath hold time for a 2-slice MB-DIR-RADFSE experiment is longer compared to a single slice DIR-RADFSE. If we assume a heart rate of 70 bpm, the minimum breath hold time for acquiring 160 views per slice with the 2-slice MB-DIR-RADFSE is ~ 18 s. Two slices acquired with the single slice DIR-RADFSE could be acquired in two 9 s breath holds. Further improvements in the MB-DIR-RADFSE would lead to shorter breath holds. For instance, the incorporation of a bent trajectory [40] or a bipolar gradient readout [41], where two radial views are acquired during each TE readout, will accelerate scan time.

Since MB encoding is performed by modulating the phase of the magnetization by the entries of a Hadamard matrix, the CPMG condition is not violated by using slab-selective refocusing pulses. However, the number of excited slices is restricted to be a power of 2. The peak power requirements of a MB RF pulse scales with the number of simultaneously excited slices. Since the proposed pulse sequence only replaces the excitation RF pulse with a MB pulse, there is a minimal increase (11%) in the time averaged RF power and specific absorption rate of the MB2 pulse sequence when compared to the single slice excitation.

Since MB-DIR-RADFSE is a black-blood imaging technique, the use of a thicker selective IR pulse would result in imperfect blood nulling in regions with slow blood flow. Thus, areas of slow flow will be bright at latter TEs and show higher T2 values compared to myocardium.

This study only considers the case of exciting two slices within a 21 mm slab. While the proposed approach could be extended for higher slice coverage, it would result in an increased scan time that would not fit in a breath hold. However, the use of prospective navigators would allow increasing the slice acceleration by acquiring data under free breathing conditions. One limitation of this study is that it does not evaluate the flow suppression performance when using larger excitation slabs and at varying flow rates. Such an evaluation would be useful in determining the maximum possible slice gap between the slices without compromising black blood contrast to noise ratio.

Finally, the iterative algorithms used for data reconstructions are computationally intensive. However, the design of optimal GPU based implementations would enable the use of iterative reconstruction algorithms in clinical settings.

## Conclusions

A technique for concurrently improving the slice and SNR efficiency of the double inversion FSE pulse sequence via MB excitation is presented. Due to the simultaneous slice excitation in MB-DIR-RADFSE, black-blood images are acquired at the null point of blood for both slices. As shown in this work, using a radial trajectory and a joint reconstruction framework has the advantage of yielding TE images with high spatial and temporal resolution for each of the slices from which a T2 map is generated for the quantitative assessment of pathologies, such a myocardial edema.

## Abbreviations

ADMM: Alternating direction method of multipliers
bSSFP: Balanced steady state free precession
CMR: Cardiovascular magnetic resonance
DIR: Double inversion recovery
ETL: Echo train length
FOV: Field of view
FSE: Fast spin echo
LGE: Late gadolinium enhancement
LV: Left ventricle/left ventricular
MB: Multi-band
MB-DIR-RADFSE: Multiband encoded double inversion recovery radial FSE
NS: Non-selective
PC: Principal component
POMP: Phase offset multi-planar
RADFSE: Radial fast spin echo
RF: Radiofrequency
ROI: Region of interest
SEPG: Slice resolved echo phase graph
SNR: Signal-to-noise ratio
SPAIR: Spectral attenuated inversion recovery
SS: Slice selective
T2-prep bSSFP: T2-prepared balanced steady-state free precession
TE: Echo time
TR: Repetition time
